# Utilizing mollusk soft tissue and shells as biomarkers for monitoring heavy metal pollution in mangrove forests

**DOI:** 10.1016/j.mex.2023.102281

**Published:** 2023-07-10

**Authors:** Kumar Krishnan, Elias Saion, M.K. Halimah, Chee Kong Yap

**Affiliations:** a Inti International University, Persiaran Perdana BBN, 71800 Nilai, Darul Khusus, Negeri Sembilan, Malaysia; b Department of Physics, Faculty of Science, University Putra Malaysia, 43400 UPM Serdang, Selangor Darul Ehsan, Malaysia; c Department of Biology, Faculty of Science, University Putra Malaysia, 43400 UPM Serdang, Selangor Darul Ehsan, Malaysia

**Keywords:** Neutron activation, Biota, Toxic metals, Mangrove snails, Biomonitoring, Biomarkers for Monitoring Heavy Metal Pollution

## Abstract

The primary objective of the study was to examine the distribution of various elements, namely Cadmium (Cd), Copper (Cu), Iron (Fe), Nickel (Ni), and Lead (Pb), in the soft tissues, shells, and associated surface sediments of *Cerithidea obtusa* (*C. obtusa*) mangrove snails collected from Sungai Besar Sepang. To conduct the analysis, the preferred and most convenient methods employed were Instrumental Neutron Activation Analysis (INAA) and Atomic absorption spectrometry (AAS). The results showed that the mean concentration of elements in the sediments and soft tissues followed the order Fe > Cu > Ni > Pb > Cd, while for the shell of *C. obtusa*, it was Fe > Ni > Cu > Pb > Cd.

• Iron (Fe) showed the highest concentration among all elements monitored in sediments, soft tissues, and shells of *C. obtusa*.
• The PF results indicated higher incorporation of Pb and Ni into shells.
• BSAF results showed that *C. obtusa* shells accumulated more Cu and Cd from sediments, making them effective biomonitors.

## Graphical abstract

**Figure:**
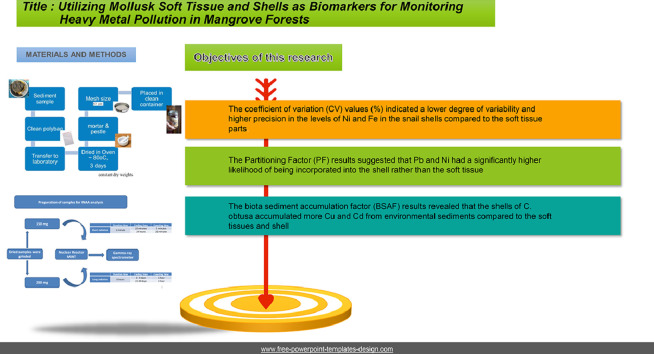

Specifications table

**Table:** 

Subject area:	Environmental Science
More specific subject area:	*Pollution*
Name of your method:	Biomarkers for Monitoring Heavy Metal Pollution
Name and reference of original method:	C. K. Yap et al., ``Shells of Intertidal Mudflat Snails: A Promising Biomonitoring Materials of Nickel Pollution,'' *Environmental Protection Research*, pp. 1–9, Nov. 2021, doi: https://doi.org/10.37256/epr.2120221052.
Resource availability:	*The data are available in this article.*

## Introduction

**Fig. 1:**
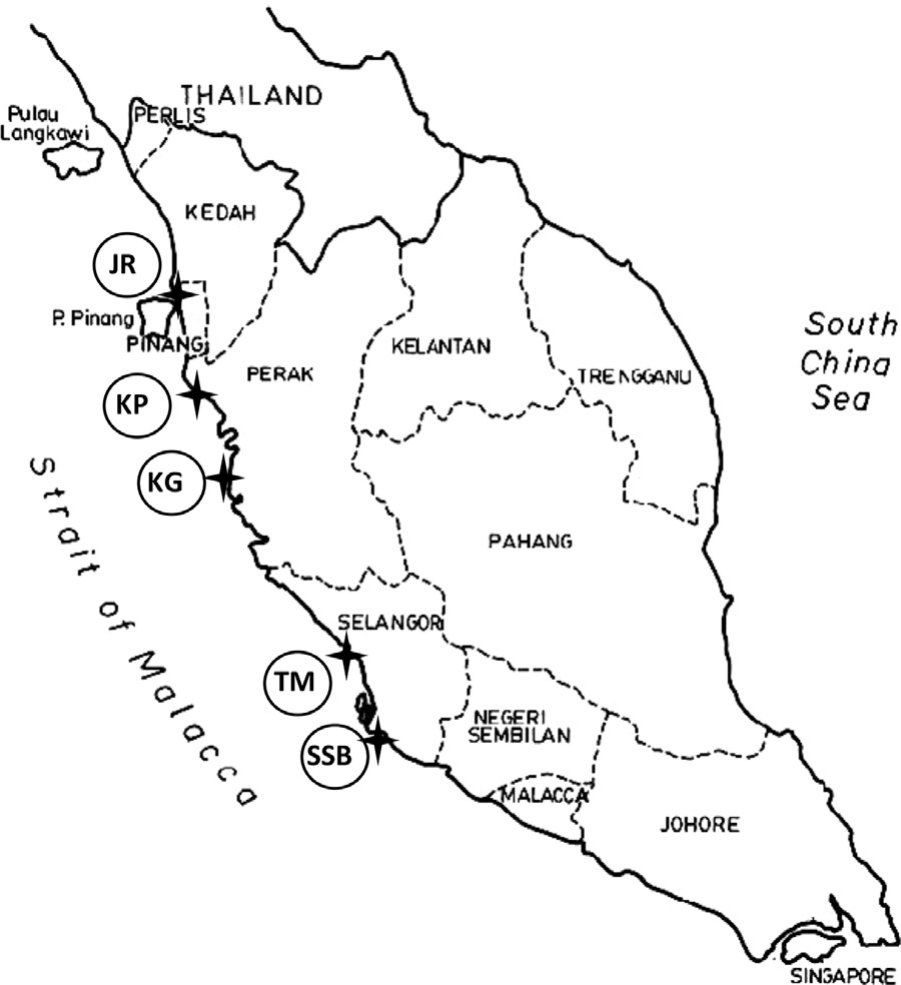
Location of the sampling stations.

Mangroves, as a unique and vital coastal ecosystem, exhibit remarkable adaptability to saline environments [1]. Within these diverse ecosystems, various species, including gastropods, thrive. Gastropods, a prominent group of mollusks found in mangroves, play crucial roles in this ecosystem by contributing to nutrient cycling, sediment stabilization, and serving as a food source for other organisms [2]. However, these delicate interactions can be affected by pollution from heavy metals. Understanding the dynamics between mangroves, gastropods, and the impacts of heavy metal pollution is essential for the preservation and conservation of these invaluable coastal ecosystems [3] (Fig. 1).

## Method details

### Sample collection and sample preparation

**Fig. 2:**
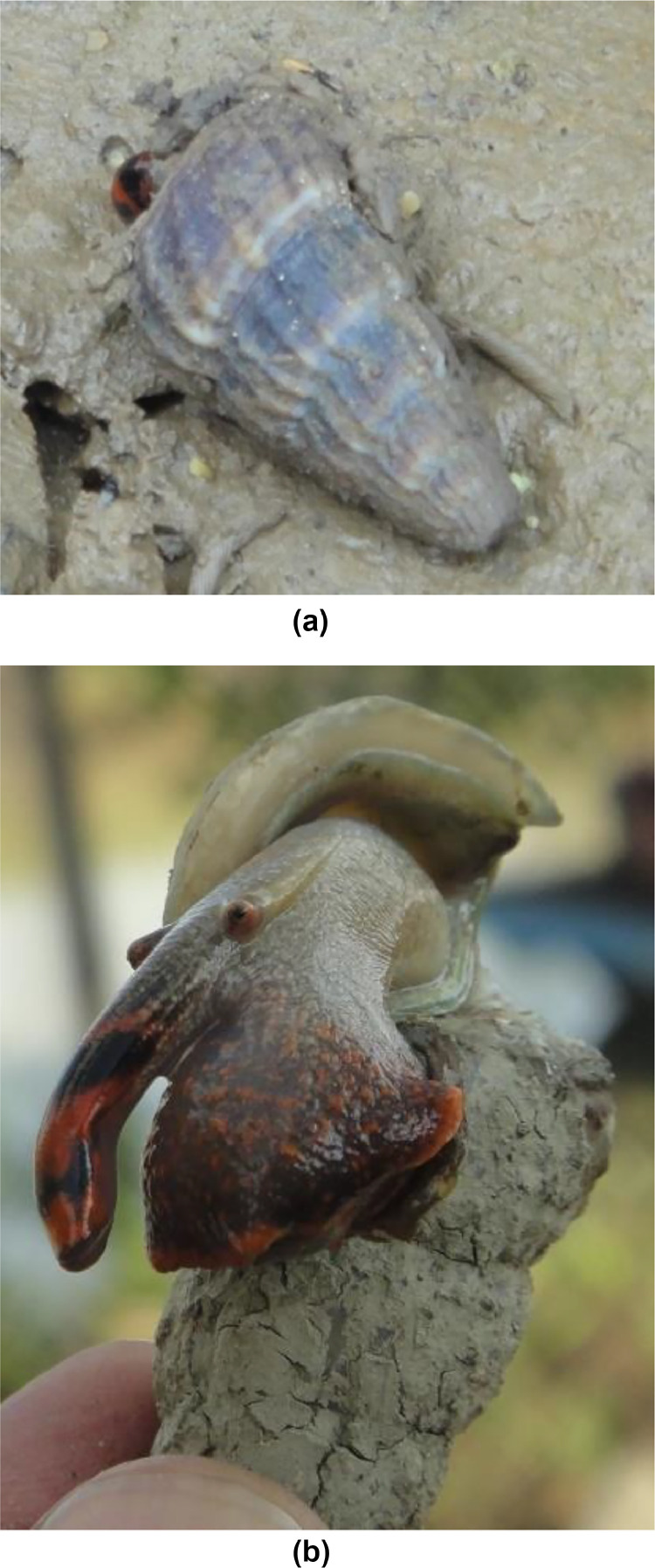
(a) Habitat of mollusk. (b) Morphology of *C. obtusa*.

Along the northern coastal intertidal zones of the west coast of Peninsular Malaysia, five stations were chosen for this study. Table 1 provides specific information regarding the GPS coordinates and activities conducted at each sampling location. The gastropod species *Cerithidea obtusa* (*C. Obtusa*) served as the focal species in this investigation. Fig. 2(a) and (b) in the accompanying materials illustrate the external morphology of both the shell and soft tissues of *C. Obtusa*. To minimize potential environmental variations and sample bias, approximately 40 °C. The *C. Obtusa* specimens of similar sizes (approximately 5 cm in length) were collected from each sampling site. To examine the potential relationship between snail characteristics and surface sediment, sub-surface sediment samples from the mangrove were gathered at a depth approximately 5.0 cm, with an approximate weight of 500–600 g [4]. At each sampling station, four surface sediment samples were gently scraped from the top layer using a clean plastic spoon. All collected surface sediments, shells, and tissues from each sample were individually placed in separate polyethylene plastic bags and stored in an ice box before being transported to the laboratory. The samples were then stored at −20 °C until further analysis. Prior to examination, the frozen *C. Obtusa* specimens were partially thawed at room temperature by scattering them on paper towels. In the laboratory, the soft tissues of *C. Obtusa* were carefully separated from the shell. Subsequently, all sediment, soft tissue, and shell samples were dried in an oven at 80 °C for 72 h or until reaching a constant dry weight (dwt). The dried samples were then powdered using a glass mortar, sieved through a mesh with a particle size not exceeding 200 µm, and stored in polyethylene pillboxes [5]*.*

**Table 1:** The description of sampling stations.

ID	Location name	GPS reading	Description of nearby activities
TM	Tok Muda, Kapar, Selangor	N 03° 7′ 30.9″ E 101° 20′ 27.7″	Residential Area, Hydroelectric, Power Plant
SSB	Sungai Sepang Besar, Selangor	N 02° 56′ 16.9″ E 101° 45′ 9.4″	Residential Area, Intertidal Area
KG	Kuala Gula, Perak	N 04° 55′ 58″ E 100° 27′ 33.6″	Fishing Village, Tourism Spot, Fish cage, shrimp pond
JR	Juru, Penang	N 05° 20′ 24.7″ E 100° 24′ 25.2″	Fishing Village, Residential Area, Industrial Area
KP	Kampung Panchor, Pantai Remis, Perak	N 04° 31′ 33.4″ E 100° 39′ 17.5″	Fishing Village, Tourism Spot, Fish cage, shrimp pond

### Analysis of heavy metals

Achieving precise analysis of samples using neutron activation analysis (NAA) and atomic absorption spectrometer (AAS) heavily depends on thorough sample preparation.

In the present study, the analysis samples were subjected to four replicate experiments, with each experiment involving the weighing of approximately 150 mg for short irradiation and 200 mg for long irradiation. The concentration of elements was determined using the Instrumental Neutron Activation Analysis (INAA) comparative method. A certified multi-element comparator, IAEA-Soil-7, was employed for calibration and quality control purposes. The irradiation process was carried out at the MINT TRIGA Mark II research reactor operating at 750 kW, utilizing a pneumatic transport facility with a thermal neutron flux of 3–4 × 10^12^ n cm^−2^ s^−1^. To ensure accuracy, the gamma spectroscopy system was calibrated using standard point sources of ^133^Ba, ^60^Co, ^57^Co, ^137^Cs, ^54^Mn, and ^241^Am within an energy range of 60 to 2 MeV, following the same geochemical configuration. For short irradiation, the samples were exposed to radiation for 1 min and then counted for 5 and 20 min after cooling for 20 min and 24 h, respectively. On the other hand, for long irradiation, the samples were irradiated for 6 h and subsequently counted for 1 hour after a cooling time of 3–4 and 21–28 days. The distance between the sample and detector was maintained at 12–15 cm for short radiation and 1–2 cm for long radiation, based on the activity level of the irradiated samples. To ensure accuracy, the dead time during counting was kept below 10% [6].

Elemental concentrations were determined using a comparative approach, and the results were expressed in terms of dry weight (d.w.). The concentration of both the sample and the Standard Reference Material (SRM) were measured using the equation [30]:

$$C_{sam}=\frac{A_{sam}}{A_{std}}x\frac{W_{std}}{W_{sam}}x\;C_{std}$$

where Asam, net count of the selected peak area of an interested element in a sample; Astd, net count of the selected peak area of an interested element in a standard; Wsam, Sample weight; Wstd, Standard weight; Cstd, Concentration of interested element in standard; Csam, Concentration of interested element in sample.

To prepare the samples for analysis, a portion weighing 1 g of each dried sample was carefully weighed and placed in a hot block digester. Concentrated HNO_3_ (AnalaR grade, BDH 69%) was used for the digestion process. Initially, the digestion took place at a low temperature of 40 °C for 1 h, followed by a high temperature of 140 °C for a minimum of 3 h. Subsequently, the digested samples were diluted using distilled deionized water (DDW). After filtration through Whatman No. 1 filter paper, the concentrations of Cd, Cu, Ni, and Pb in the prepared samples were determined using an atomic absorption spectrophotometer (AAS). The results were expressed in milligrams per kilogram (mg kg^-1^) of sample dry weight [7].

The Statistical Program for Social Science (SPSS) for Windows (Version 15) was employed to evaluate the relationships between Ni, Cd, Fe, Cu, and Pb levels in the sediment and different parts of the snails (shell and soft tissue) using correlation coefficients (CA) and multiple linear stepwise regression analysis (MLSRA). Prior to conducting the statistical analysis, a logarithmic transformation (log10(mean + 1)) was applied to all data for the CA and MLSRA analyses. This transformation was implemented to reduce the variance and ensure a more robust analysis [8].

The coefficient of variation (CV) value serves as a measure of variability for parameters [9], quantifying the dispersion of data in relation to their respective standard deviation. If the standard deviation increases, it indicates a higher level of variability in the parameter under investigation [10]. To calculate the CV value, the untransformed data was used, and it was determined using the following Eq. (1):

$$\text{CV}\;\left(\%\right)\;=\frac{standard\;deviation}{mean}\;x\;100$$

The bioaccumulation factors (BSAF) value was determined for the various tissues of the snails using Eq. (2):

$$\text{BSAF}\;=\;\frac{mean\;of\;X\;concentration\;in\;the\;tissue}{mean\;of\;X\;concentration\;in\;the\;associated\;sediment}\;x\;100$$

This calculation enabled the assessment of the extent to which certain substances accumulated in the tissues of the snails in relation to the concentration of those substances in the environmental samples.

In accordance with Dallinger's classification [11], the BSAF values can be categorized into three groups: ``deconcentrators'' when the BSAF is less than 1, ``microconcentrators'' when the BSAF falls between 1 and 2, and ``macroconcentrators'' when the BSAF exceeds 2. This classification provides a framework for evaluating the degree of bioaccumulation of substances in the tissues of the snails based on their BSAF values.

## Results and discussions

The analytical results for the certified reference material and the measured values of SL-1, SRM 1566b, and SRM 2976 for major elements are presented in Tables 2 and 3. The accuracy of the analytical procedure was assessed through a recovery test, which yielded excellent results. The percentage recovery values ranged from 82.1% to 144.6% for SL-1 and 92.3% to 141.2% for SRM 1566b/SRM 2976. These findings demonstrate the reliability and precision of the analytical method employed, indicating its ability to accurately determine the concentration of major elements in the samples.

**Table 2:** Analysis of the measured concentration of CRM of SL-1 value compared with certified reference materials.

Element	Certified Value	Measured Value	Recovery (%)
**Fe**	67,400	67,916	100.8
**Cu**	11	11.14	101.3
**Cd**	1.3	1.88	144.6
**Ni**	26	28.15	108.3
**Pb**	60	49.25	82.1

**Table 3:** Analysis of the measured concentration of CRM of *SRM 1566b and SRM 2976 value compared with certified reference materials.

Element	Certified value	Measured value	Recovery (%)
**Fe**	205.8	263.7	128.1
**Cu**	71.6	70.1	97.9
**Cd**	0.82	0.85	103.7
**Ni**	0.93	0.86	92.3
**Pb**	1.19	1.68	141.2

The concentrations of Fe, Cu, Cd, Ni, and Pb in the soft tissue and shell of *C. Obtusa*, along with their associated sediments, are presented in Table 4. The study revealed that the Fe concentration in the sediment ranged from 9052 to 34,579 mg/kg, with a mean of 21,187 mg/kg. In comparison, the Fe concentration in the tissues ranged from 652.4 to 5755 mg/kg, with a mean of 2872 mg/kg, while in the shell, it ranged from 316 to 403 mg/kg, with a mean of 362 mg/kg. Notably, the sediment exhibited a higher Fe concentration than both the tissues and shell of C. *Obtusa*. When comparing these findings to other studies (Table 4), it was observed that the Fe concentration in this study was higher (Author et al., Year). Before 2015, the establishment of maximum limits for Fe by international organizations such as the World Health Organization (WHO), Food and Drug Administration (FDA), and Food and Agriculture Organization (FAO) was limited. The Joint FAO/WHO Expert Committee on Food Additives (JECFA) had previously expressed doubts regarding the maximum tolerable level of Fe, as acknowledged by a global panel of experts [12]. Therefore, comparing the Maximum Permissible Limits (MPLs) with the available Fe data is deemed impossible, given the uncertainty surrounding Fe limits [12].

**Table 4:** Concentration of heavy metals (mg/kg) in tissue, shell and associated sediment.

	Element\Location	TM	SSB	KG	JR	KP
Sediment	Fe	13,972 ± 1848 (12,666,16,669)	23,659 ± 1910 (21,874,25,674)	24,675 ± 1215 (23,191,26,076)	34,579 ± 1797 (33,405,37,232)	9052 ± 448 (8622,9495)
	Cu	5.70 ± 0.69 (4.98,6.37)	42.66 ± 3.88 (38.10,47.23)	84.73 ± 4.55 (78.88,88.77)	82.21 ± 4.62 (78.85,89.04)	64.96 ± 6.64 (57.80,73.85)
	Cd	0.270 ± 0.057 (0.217,0.330)	nd	0.521 ± 0.036 (0.488,0.560)	0.639 ± 0.063 (0.572,0.696)	0.436 ± 0.046 (0.392,0.484)
	Ni	23.88 ± ± 3.59 (20.29,27.46)	22.72 ± 5.48 (17.23,28.20)	23.62 ± 3.62 (20.00,27.23)	33.76 ± 6.00 (27.76,39.75)	17.46 ± 6.22 (11.23,23.6)
	Pb	27.04 ± 5.50 (23.14,30.93)	15.41 ± 7.51 (10.35,24.04)	27.16 ± 3.71 (23.71,31.08)	34.52 ± 9.41 (27.64,45.25)	13.16 ± 3.74 (9.048,16.38)
Tissues	Fe	2230 ± 2236 (224,4822)	5755 ± 3682 (2066,9431)	652 ± 41 (606,705)	4205 ± 116 (4102,4369)	1517 ± 84 (1449,1630)
	Cu	102.66 ± 0.79 (102.10,103.22)	97.24 ± 9.40 (90.59,103.88)	46.94 ± 7.59 (41.58, 52.30)	139.96 ± 24.70 (122.50,157.43)	161.44 ± 18.70 (148.22,174.66)
	Cd	0.148 ± 0.042 (0.108,0.192)	0.773 ± 0.132 (0.640,0.904)	0.436 ± 0.047 (0.396,0.488)	0.859 ± 0.073 (0.792,0.936)	0.030 ± 0.006 (0.023,0.034)
	Ni	19.79 ± 4.27 (15.52, 24.06)	18.19 ± 0.22 (17.97,18.42)	18.33 ± 0.38 (17.95,18.70)	23.35 ± 2.81 (20.54,26.16)	15.35 ± 2.96 (12.39,18.31)
	Pb	5.63 ± 1.24 (4.54,6.98)	5.03 ± 1.59 (4.06,6.86)	3.00 ± 1.23 (1.60,3.90)	9.28 ± 1.81 (7.28,10.82)	6.13 ± 5.23 (1.27,11.66)
Shell	Fe	380.8 ± 18.1 (357.9,401.2)	315.9 ± 41.5 (286.6,345.3)	394.7 ± 86.5 (313.5,505.5)	402.8 ± 70.5 (339.0,490.4)	315.6 ± 59.1 (238.1,371.5)
	Cu	13.38 ± 1.61 (12.51,14.79)	2.11 ± 0.54 (1.73,2.50)	8.62 ± 3.62 (6.06,11.18)	4.61 ± 2.42 (2.90,6.32)	3.66 ± 3.02 (1.52,5.79)
	Cd	0.305 ± 0.033 (0.272,0.338)	0.327 ± 0.014 (0.311,0.336)	nd	0.551 ± 0.047 (0.512,0.603)	0.145 ± 0.008 (0.139,0.154)
	Ni	15.66 ± 1.90 (13.76,17.56)	14.88 ± 2.90 (11.98,17.78)	13.80 ± 5.87 (7.94,19.67)	14.33 ± 1.88 (12.45,16.22)	12.60 ± 6.84 (5.76,19.43)
	Pb	5.66 ± 1.54 (4.57,6.74)	6.33 ± 2.25 (4.74,7.92)	8.06 ± 0.03 (8.04,8.08)	9.26 ± 1.56 (8.16,10.36)	5.13 ± 2.54 (3.34,6.93)

The concentration of Cu in the sediments of *C. Obtusa* exhibited a range of 5.7 to 84.7 mg/kg, with a mean concentration of 56.1 mg/kg. In comparison, the Cu concentration in the tissues ranged from 46.9 to 161.4 mg/kg, with a mean of 109.6 mg/kg, while in the shell, it ranged from 2.11 to 13.4 mg/kg, with a mean of 6.48 mg/kg. Notably, the Cu concentration in the tissues of *C. Obtusa* was higher than that in the shell and associated sediments. Comparing the Cu accumulation in *C. Obtusa* from this study with other studies, it was observed that the Cu concentration in this study was higher than the Malaysian Maximum Permissible Limit (MPL) of 30.0 mg/kg wet weight [13]. Additionally, when comparing it to the legal limits established by the Food and Agriculture Organization (FAO) (20–70 mg/kg wet weight) [14], the Cu accumulation exceeded these limits as well. The higher accumulation of Cu in the tissues can be attributed to its incorporation into various proteins, including respiratory proteins and enzymes [[15], [16], [17]].

The concentration of Cd in the sediments of *C. Obtusa* ranged from 0.27 to 0.639 mg/kg, with a mean concentration of 0.467 mg/kg. In comparison, the Cd concentration in the tissues ranged from 0.3 to 0.773 mg/kg dry weight (0.0830 mg/kg wet weight), with a mean of 0.347 mg/kg dry weight (0.0794 mg/kg wet weight), while in the shell, it ranged from 0.145 to 0.551 mg/kg dry weight (0.0794 mg/kg wet weight), with a mean of 0.332 mg/kg dry weight (0.0794 mg/kg wet weight). Notably, the Cd concentration in the sediments was higher than that in the tissues and shell of *C. Obtusa*. When comparing the Cd concentration with regulatory limits, EU Commission Regulation 2021/1323 [19] establishes a maximum limit of 1.0 mg/kg fresh weight for Cd in bivalve molluscs. In this study, the Cd levels were well within this limit (Author et al., Year). Furthermore, the comparison was made against the Malaysian Food Regulations (MFR) Cd MPL of 1.00 mg/kg wet weight, the Commission Regulation of the European Union, which sets a limit of 2.00 mg/kg wet weight for Cd in marine bivalves, and the MPLs (1–2 mg/kg wet weight) compiled by FAO for Cd in fish/fish products/shellfish. The results from this study were lower than these limits. Additionally, the Cd levels were also lower compared to other studies [15,18,19].

The concentration of Ni in the sediments of *C. Obtusa* ranged from 17.5 to 33.8 mg/kg, with a mean concentration of 24.3 mg/kg. In comparison, the Ni concentration in the tissues ranged from 15.4 to 23.4 mg/kg, with a mean of 19.0 mg/kg, while in the shell, it ranged from 12.6 to 15.7 mg/kg, with a mean of 14.3 mg/kg. Notably, the Ni concentration in the sediments was higher than that in the tissues and shell of *C. Obtusa*. Regulatory Maximum Permissible Limits (MPLs) for Ni are not commonly found in the literature [20]. However, the US Food and Drug Administration (FDA) has established the only accessible Ni MPL, referred to as the action level, which is set at 80 mg/kg wet weight (333.3 mg/kg dry weight) [21]. The Ni levels in all populations of *C. Obtusa* in this study were significantly below the Ni action limit, indicating that there is no non-carcinogenic risk associated with Ni consumption from snails.

The concentration range of Pb in *C. Obtusa* was 13.2–34.5 mg/kg (mean: 23.5 mg/kg). In tissues, it ranged from 3.00 to 9.28 mg/kg (mean: 5.81 mg/kg), and in the shell, it ranged from 5.13 to 9.26 mg/kg (mean: 6.89 mg/kg; 1.43 mg/kg difference). Sediment exhibited higher Pb levels than tissues and shell. Compared to the Malaysian MPL (2.00 mg/kg ww [13]), *C. Obtusa* had lower Pb concentrations, with 38% of populations below the Pb MPL (Fig. 2). Comparable conclusions can be drawn when comparing Pb safety guidelines with those proposed by the European Union (1.50 mg/kg ww), US Food and Drug Administration (1.70 mg/kg ww [21]), and ANZFA (2.00 mg/kg ww). FAO's Pb legal limits (2.00–10.0 mg/kg ww) were also considered [13,14,21].

Based on data in Table 5, the coefficient of variation (CV) values (%) for Fe, Cd, Pb, Ni, and Cu in the total soft tissues were 72.3, 81.8, 39.1, 15.3, and 40.1, respectively. In contrast, the CV values in the total shells were 11.9 for Fe, 50.3 for Cd, 25.1 for Pb, 8.1 for Ni, and 70.2 for Cu. These findings suggest that Ni and Fe levels in the shells exhibit lower variability and higher precision compared to the soft tissue parts of the snails. The reduced variability of shell metals may be attributed to dissimilarities in biochemical behavior and biological half-lives of Ni between mollusk shells and their soft tissue parts [22,23].

**Table 5:** Comparison between coefficients of variation (%) of heavy metal concentrations in the soft tissues and the shells of *S. cucullate* collected from the northern Coast of Qeshm Island on the Persian Gulf of Iran.

Different part	Fe	Cu	Cd	Ni	Pb
Soft tissue	72.3	40.1	81.8	15.3	39.1
Shell	11.9	70.2	50.3	8.1	25.1

In the present study, the snails' sizes (shell heights and widths) were measured at 5.0 cm and 2.5 cm, respectively. The partitioning factor (P.F.), which defines the ratio of mean metal concentrations in the soft tissue and shell, was used to analyze metal distribution [24]. P.F. values are presented in Table 6. Fe, Cu, Cd, Ni, and Pb exhibited high P.F. values ranging from 1.65 to 18.22, 5.45 to 46.09, 0.21 to 2.36, 1.22 to 1.63, and 0.37 to 1.19, respectively. These findings align with previous studies [[25], [26], [27]]. Additionally, Cu and Fe play crucial roles in mollusk soft tissue metabolism. Pb and Ni demonstrated the lowest P.F. values among the metals, suggesting a higher likelihood of their incorporation into the shell rather than the soft tissue.

**Table 6:** Partitioning factors of the gastropods from the study sites.

	Fe	Cu	Cd	Ni	Pb
TM	5.86	7.67	0.49	1.26	0.99
SSB	18.22	46.09	2.36	1.22	0.79
KG	1.65	5.45		1.33	0.37
JR	10.44	30.36	1.56	1.63	1.00
KP	4.81	44.11	0.21	1.22	1.19

The bio-sediment accumulation factor (BSAF) was determined and presented in Table 7 to assess the uptake capacity of heavy metals from environmental sediments in both the shells and tissues. The majority of heavy metals classified the shell and soft tissue of the snails as `deconcentrators,' with the exception of Cu and Cd. In the case of snails collected from sampling location TM, the shells were classified as `microconcentrators' for Cd and Cu. These BSAF findings suggest that *C. Obtusa* shells accumulate more Cu and Cd from environmental sediments compared to soft tissues and shells. The discrepancy in results can be attributed to species digestion physiology and metal absorption rates [28,29,31,32].

**Table 7:** BSAF-SH (shell) and BSAF-ST (soft tissue).

	BSAF SH		ST				BSAF			
	Fe	Cu	Cd	Ni	Pb	Fe	Cu	Cd	Ni	Pb
TM	0.03	2.35	1.13	0.66	0.21	0.16	18.01	0.55	0.83	0.21
SSB	0.01	0.05		0.65	0.41	0.24	2.28		0.80	0.33
KG	0.02	0.10	0.00	0.58	0.30	0.03	0.55	0.84	0.78	0.11
JR	0.01	0.06	0.86	0.42	0.27	0.12	1.70	1.34	0.69	0.27
KP	0.03	0.06	0.33	0.72	0.39	0.17	2.49	0.07	0.88	0.47

The correlations between metals in soft tissues, the shell of *C. Obtusa*, and sediments are presented in Table 8 (Pearson's correlation coefficients). With the exception of Cu, heavy metal concentrations in surface sediments exhibited significant positive correlations with corresponding metals in the soft tissues and shell of *C. Obtusa*. Notably, Fe, Pb, Ni, and Cd concentrations in soft tissues displayed strong associations with the metal levels in the surrounding sediments, indicating the ability of bivalve mollusks to reflect environmental metal concentrations. Additionally, substantial relationships were observed between the shells and surface sediments for Fe, Pb, Ni, and Cd, suggesting the potential use of *C. Obtusa* shells as biomonitoring indicators for these four metals.

**Table 8:** Correlation between element concentrations in surface mangrove, tissues and Shell of *C. otusa*.

Fe			
	Soft tissue	Shell	Sediment
Soft tissue	1		
Shell	0.921	1	
Sediment	0.942	0.976	1

Cu			
	Soft tissue	Shell	Sediment
Soft tissue	1		
Shell	−0.41364	1	
Sediment	−0.00452	−0.50556	1

Pb			
	Soft tissue	Shell	Sediment
Soft tissue	1		
Shell	0.320586	1	
Sediment	0.372875	0.790399	1

**Ni**			
	Soft tissue	Shell	Sediment
Soft tissue	1		
Shell	0.541361	1	
Sediment	0.981101	0.395289	1

Cd			
	Soft tissue	Shell	Sediment
Soft tissue	1		
Shell	0.822199	1	
Sediment	0.826268	0.646599	1

## Conclusion

The mudflat snail, *C. Obtusa*, is a frequently encountered species in intertidal areas, renowned for its ease of identification and collection, rendering it highly suitable for ecotoxicology studies. The research findings indicate that the shells of *C. Obtusa* hold significant potential as biomonitoring materials for evaluating Cu and Cd pollution in intertidal regions. These discoveries underscore the significance of ongoing monitoring of heavy metal discharges from human activities and the implementation of more stringent environmental protection measures. However, additional validation is necessary through experimental studies conducted under both field and laboratory conditions.

## CRediT authorship contribution statement

**Kumar Krishnan:** Conceptualization, Methodology, Software, Validation, Formal analysis, Investigation, Data curation, Writing – original draft. **Elias Saion:** Conceptualization, Writing – review & editing, Supervision. **M.K. Halimah:** Conceptualization, Writing – review & editing, Supervision. **Chee Kong Yap:** Conceptualization, Writing – review & editing, Supervision.

## Declaration of Competing Interest

The authors declare that they have no known competing financial interests or personal relationships that could have appeared to influence the work reported in this paper.

## Data Availability

The data that has been used is confidential.
